# Bone healing of severe acetabular defects after revision arthroplasty: A clinical positron emission tomography study of 7 cases

**DOI:** 10.3109/17453670902947416

**Published:** 2009-04-01

**Authors:** Gösta Ullmark, Jens Sörensen, Olle Nilsson

**Affiliations:** ^1^Department of Orthopedics, Gävle Hospital Center for Research and Development Uppsala University/Gävleborg County CouncilUppsalaSweden; ^2^Department of Nuclear Medicine, Uppsala University HospitalUppsalaSweden; ^3^Department of Orthopedics, Uppsala University HospitalUppsalaSweden

## Abstract

**Background and purpose** Healing of acetabular bone grafts may be difficult to assess in conventional radiographs. We used PET to analyze healing of morselized bone allografts, impacted in large osteolytic acetabular defects at revision arthroplasty.

**Patients and methods** 7 cases had a cup revision because of loosening, with repair of a segmental defect using a perforated, wide and thin plate. The osteolytic acetabulum was impacted with morselized bone allograft before cementing a polyethylene cup. [^18^F]-fluoride PET scans were used to monitor bone healing inside the graft bed 1 week, 4 weeks, and 12 months after surgery. The contralateral pelvic bone above the acetabulum was used as reference. A second group of 4 cases was analyzed for bone-forming activity in the state of mechanical loosening of an acetabular component of a THA.

**Results** Preoperatively, the uptake was raised by 64% compared to the reference. 1 week after surgery it was increased by 77% in segmental regions, while the uptake was at the reference level in cavitary regions. After 4 months the uptake was increased by 91% in cavitary regions and by 117% in segmental regions. 1 year after surgery, the increase in uptake was 20% in both regions. All implants were stable on radiographs.

**Interpretation**We found PET to be a sensitive and useful method for evaluation of the spatial and temporal development of bone formation.

## Introduction

Cup loosening often leads to bone defects. The defects can be graded into cavitary (with an enlarged acetabular cavity but with an intact bony rim), segmental (where some part of the acetabular rim is absent), and pelvic discontinuity. Often there are combinations of cavitary and segmental defects of type III (AAOS classification). In the US, it has been a general tradition to use structural allografts for combined defects together with either cemented (Jasty and Harris 1990) or uncemented cups (Chandler 1992, Hooten et al. 1996, Shinar and Harris 1997). Structural allograft is associated with the problem of late graft failure (Mulroy and Harris 1990). The structural graft may also be combined with a reinforcement ring and a cemented cup (Gill et al. 2000). An uncemented cup with screws combined with morselized bone graft is an alternative. This method was recently reported with 6/47 repeat revisions after a mean of 5 years follow-up (Tze-Pui and Kwong-Yuen 2003). In Europe, the predominant method for treatment of large acetabular defects in prosthetic loosening is impacted morselized allograft supported by a reinforcement ring (Udomkiat et al. 2001) or supported by a metal mesh, together with a cemented cup (Schreurs et al. 2001).

Positron emission tomography (PET) can be used to study metabolic events in vivo, and has been established as major research tool in many areas of life science. So far, however, PET applications in orthopedics are few (Piert et al. 1999, Sörensen et al. 2003, Ullmark et al. 2007). Radioactive isotopes are administered and detected using a tomographic approach that allows correlation to anatomical regions. Various molecules such as proteins and ions can be marked by isotopes and associated metabolic events can thus be detected. Thus, the tomographic images produced represent a metabolic event that can subsequently be correlated to an anatomical region. In the case of fluoride PET, this ion is metabolized into the bone mineral and can be used to produce quantitative images that correlate with new bone formation (Sörensen et al. 2003, Ullmark et al. 2007).

We analyzed the course of bone healing in the impacted graft beds in the acetabulum, using [^18^F]-fluoride PET. We also describe the surgical method of restoring the deficient acetabulum with flexible perforated titanium plates, combined with preparation and impaction of fresh frozen morselized bone allograft.

## Patients and methods

### Patient

One group of 7 hips (6 patients, 3 females) with ages ranging from 55 to 77 years was included in this study. All patients had a mechanical loosening of a primary THA together with loss of acetabular bone. All patients had both segmental and cavitary bone defects of type III (AAOS classification). One of the patients had undergone 1 previous revision THA. The primary THA had been performed due to osteoarthritis in 5 hips and rheumatoid arthritis in 2. The contralateral hip was healthy without any previous surgery in 4 cases, while 2 cases had a well-performing THA. The surgical procedure was performed by one surgeon (GU) who was well acquainted with the surgical technique. In all 7 cases, the femoral component was also revised to a distal anchoring, tapered and uncemented stem.

A second group of 4 matched cases (2 females) with ages ranging from 62 to 80 years and with mechanical loosening and acetabular osteolysis of a THA, were PET-scanned for bone metabolism in the osteolytic acetabular region, and used as an osteolytic reference. The diagnosis was osteoarthritis for 3 of these patients and rheumatoid arthritis for 1.

### Bone graft

Bone grafts were taken from fresh frozen femoral heads harvested at primary arthroplasty for hip osteoarthritis and stored at –80°C. The head was cleaned of most of the cartilage and fibrous tissue. The bone was morselized with a Howex milling machine (Gävle, Sweden) that produced chips of dimension up to a maximum of 2 × 4 × 5 mm. The milled bone chips were partly defatted by repeated rinsing in warm saline solution (40°C).

### Surgical technique

**Figure 1.**
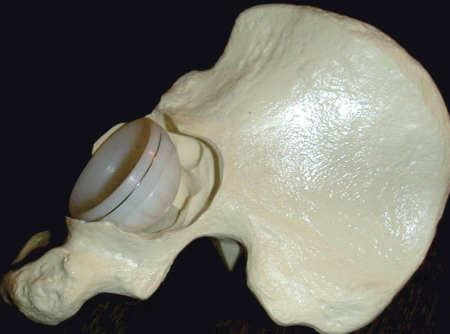
Combined segmental and cavitary osteolytic bone defects of the acetabulum.

**Figure 2.**
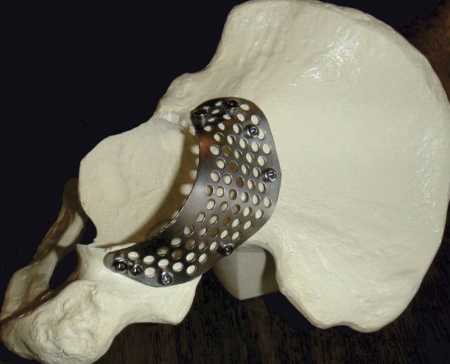
Titanium Acetabular Rim Mesh to restore a segmental lateral wall defect.

**Figure 3.**
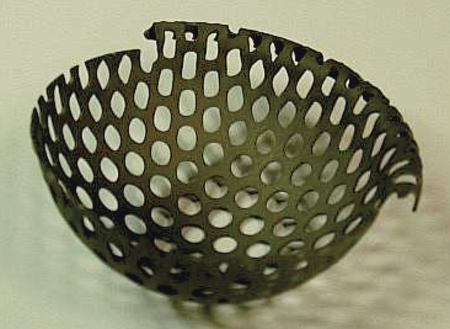
Titanium Acetabular Graft Cup.

**Figure 4.**
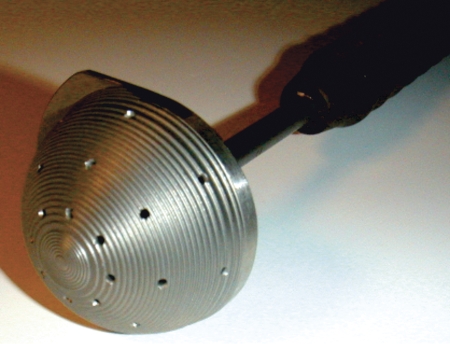
Acetabular bone graft impactor.

All patients received systemic antibiotic prophylactics during surgery. All were operated using a posterior surgical approach. The loose prosthesis and cement was removed together with any debris and fibrous membrane, to lay bare the cavitary widened acetabulum. All patients also had a segmental defect of the acetabular rim (Figure 1). The rim defects were restored from the outside by a flexible 0.8-mm-thick metal plate fully perforated with 4-mm holes. This Titanium Acetabular Rim Mesh (Waldemar Link GmbH, Hamburg, Germany) was anchored with several small titanium screws (Figure 2). The medial wall defect was restored by a semi-flexible 0.8-mm thick perforated cage: a Titanium Acetabular Graft Cup from the same company (Figure 3). The cage is also perforated with 4-mm holes to allow for screw fixation, and for bone ingrown through the holes. Graft impaction was performed using acetabular impactors from the Lubinus SPII Impaction Instrument Set (Waldemar Link). Those impactors are equipped with a microstructure to enhance hard impaction (Figure 4). There are also several small holes for evacuation of liquid to produce a more stable graft bed (Fosse et al. 2006). This structure of the impactor may enhance the containment of a blood clot within the graft layer. We aimed for the true center of rotation of the hip. An acetabular polyethylene cup of 4-mm smaller diameter than the very hard impacted neo-acetabular diameter was cemented using Palacos cum gentamicin, and thus space for a 2-mm cement mantle was created.

### Postoperative care

All patients were treated with low-molecular-weight heparin for 9–21 days. They were mobilized on the first or second day after surgery to walk with toe-touch weight bearing on the operated leg, using two crutches for 6 weeks.

### Follow-up

The patients were clinically and radiographically examined 1 year after surgery. Clinical outcome was assessed using the Charnley modification of the Merle d'Aubigné-Postel classification, where 6 is normal, pain free function. PET analysis was performed 1 week, 4 months, and 1 year after surgery. 1 patient died due to unrelated causes 6 months after surgery. She was included in the 1-week PET analysis. Another patient was diagnosed to have a periprosthetic infection after 15 months. Several tissue cultures for bacterial growth taken at the time of surgery were negative. Onset of the first symptom of infection was noticed 1 year after surgery. He was subsequently included in the 1-week- and 4-month PET analyses, but was excluded from the 1-year analysis.

### PET analysis

We used a Siemens/CTI Exact HR+ scanner (Siemens/CTI, Knoxville, TN). The HR+ has a FOV of 15 cm, yielding 63 transaxial slices. Patients were placed in the supine position on the camera bed. The legs were fixed in place by a vacuum cushion. A venous catheter was inserted into an ante cubital or dorsal hand vein for injection of tracer.

30 min after intravenous injection of 200 MBq [18F]-fluoride, a section of the body from the knees to the upper pelvis was scanned in 2D whole-body mode. Emission scanning started from the knees (5 min per 15 cm bed position) and moved proximally to cover the entire hip prosthesis area in one session. Transmission scanning for attenuation correction was performed after completing the emission acquisition.

All emission scans were corrected for attenuation, scatter, and decay and reconstructed by a process of filtered backprojection. The analysis method has been described in detail elsewhere (Sörensen et al. 2003). Briefly, regions of interest (ROIs) were graphically placed at the region of segmental defect, and at the region of cavitary defect. Standardized uptake values (SUVs) from these ROIs were calculated by the formula: SUV of tissue = activity in tissue (Bq/mL) × body weight (g) / total dose injected (Bq). Setting average body density to 1 g/mL, this expression gives a unitless value of the regional tissue activity in proportion to the average activity per mL of the entire body. Average values of contralateral healthy regions of the pelvis are presented as reference values in the text.

## Results

### Clinical results

The mean (range) clinical outcomes according to the Merle d'Aubigné-Postel classification were: preoperatively, 3.9 (3–5), 3.7 (2–5), 5.1 (5–6), and at follow-up, 5.5 (5–6), 5.5 (5–6), 5.3 (5–6), respectively.

### Radiographic results

No cups or plates had migrated on plain radiographs. There were no radiolucent lines.

### Preoperative PET results in osteolytic cavitary regions (reference cases)

**Figure 5.**
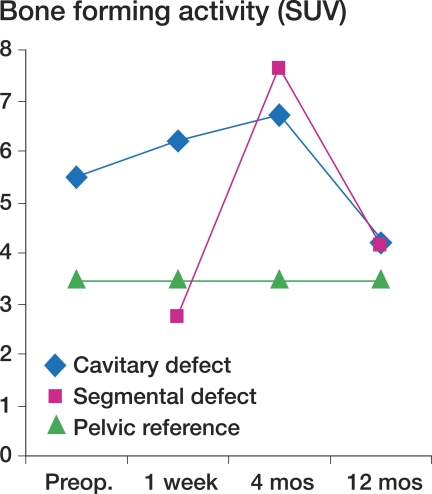
Mean values of quantitative [18F]-fluoride uptake in various regions.

In the 4 matched cases with prosthetic loosening, the uptake was raised from mean 3.5 (SD 0.8) to mean 5.5 (SD 1.5) SUV (Figure 5).

### Postoperative PET results in cavitary and segmental defect regions

1 week after surgery, the mean uptake was 6.2 (SD 1.7) SUV in cavitary defects and 2.8 (SD 0.9) in segmental defects. At 4 months, it was 6.7 (SD 2.9) in cavitary defects and 7.6 (SD 3.8) in segmental defects. At 1 year, it was 4.2 (SD 0.9) in cavitary defects and 4.1 (SD 1.3) in segmental defects.

## Discussion

Scintigraphic studies have revealed elevated technetium uptake in endoprosthetic loosening (Temmerman et al. 2005), indicating elevated bone metabolism. In the present study the fluoride uptake was highly elevated preoperatively in osteolytic areas, indicating highly elevated bone formation (Messa et al. 1993) in this predominantly osteolytic lesion. Although the net effect is an osteolytic loss of bone, a highly elevated degree of new bone formation is present at the same time in the same region. This finding probably corresponds to the local presence of activated osteoblasts and mesenchymal stem cells. Subsequently, when a loose arthroplasty has been surgically revised, the state of preoperatively elevated bone formation is still present but the osteolytic process is removed. This mechanism may be an explanation for the highly elevated fluoride uptake in cavitary defects even only 1 week after revision surgery using impaction grafting of a loosened prosthesis. In a previous PET study from our group analyzing bone healing in a similarly impacted graft bed in the proximal femur (Sörensen et al. 2003), similar findings of a swift onset of bone healing were obtained to support this explanation. In the present study, healing activity in cavitary defects was increasing continuously after 4 months. 1 year after surgery, for both cavitary and segmental defects bone formation had declined almost to the reference level.

**Figure 6.**
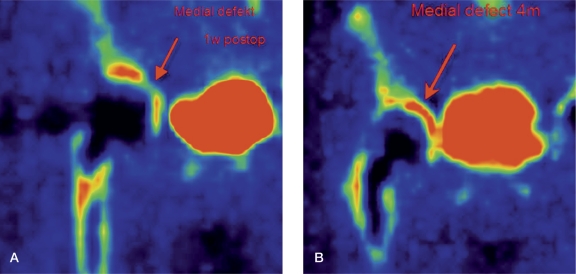
A. PET image. Arrow indicating no activity in a segmental acetabular defect 1 week after surgery. B. The same case as in panel A. An arrow indicates high activity (bone healing) in the segmental defect 4 months after surgery.

The bone grafts in the segmental acetabular defects were impacted into contact with the perforated plate, and were not in contact with remaining acetabular bone. Those regions had a low regenerative activity 1 week after surgery, but it was highly elevated after 4 months (Figure 6). This phenomenon corresponds to an initial absence of osteoblasts and mesenchymal stem cells in close contact with bone graft in segmental defects where the inner surface of such a graft bed consists of the perforated titanium plate. Behind this perforated plate, soft tissues such as muscle and fibrous tissue are present. Mesenchymal stem cells or progenitor cells may migrate from such soft tissues through the holes of the plate and into the bed of impacted bone graft. In this environment, consisting of a composite of blood clot and bone chips, a differentiation into osteoblasts may take place. The alternative way for bone-forming cells to enter the segmental graft bed is to migrate from the nearest remaining acetabular bone. Either way, a time delay in the onset of bone formation should occur in this region—as we found. After 4 months, the intensity of bone healing in the segmental defects increased to that of the cavitary defect. Our conclusion is that an impacted bed of fresh frozen, morselized and fat-reduced bone graft in contact with a perforated plate has a delayed onset but an equally intense course of bone healing compared to the same graft bed in a cavitary defect. One consequence of this finding is that a perforated plate used to convert a segmental acetabular defect to a cavitary one has to be sufficiently stable to withstand the load until bony healing has taken place in the adjacent graft bed. Our conclusion is that the plate does not obstruct bone healing. The finding of intense bone regeneration within the morselized allograft is further confirmed by the finding of good clinical outcomes and good radiographic results in all patients, with no signs of implant loosening or migration.

### Tracer used in this study

We used [^18^F]-fluoride PET to acquire an established index of regional bone metabolism, KPAT (Hawkins et al. 1992, Piert et al. 1998). KPAT defines the rate at which ^18^F-fluoride ions are extracted from plasma and irreversibly deposited in bone mineral. This is an active and complex process, believed to correspond to osteoblastic activity (Schiepers et al. 1997). In a recent study (Piert et al. 2001), kinetic ^18^F-fluoride PET was compared to bone formation rate in pigs as assessed by invasive bone histomorphometry, and a significant correlation was found. We also calculated a semiquantitative estimate of regional fluoride uptake, in the PET nomenclature called standard uptake value (SUV). This type of measurement is often used in clinical PET applications because it can be calculated from a single tomographic image and it does not require blood sampling. We have previously shown that there is a high correlation between SUV measurements and KPAT values in the hip and femoral regions (Sörensen et al. 2003), thus providing an opportunity for simplified scanning.

A discovery of considerable interest regarding fresh frozen bone allograft is described in a recent in vitro study by Board et al. (2008). Strain, as from vigorous graft impaction and postoperative load, was shown to transform bone homograft from osteoconductive to osteoinductive, since BNP-7 was found to be released from the graft in proportion to the strain imposed on it.

In summary, our findings indicate that the impacted bone allograft had transformed to living bone, confirming the findings of Piert et al. (1999). They found in a fluoride-PET study after acetabular revision of THA and impaction of morselized allograft that the bone metabolism in the former graft bed did normalize over time. These favorable clinical results are supported by the good results of bone graft transforming to living bone reported in a clinical study of 30 cases (Ullmark and Wojdyna 2003). The patients had combined acetabular defects (type III, AAOS) and were analyzed mean 7 years after the same type of revision THA, including impaction grafting and restoration of segmental defects by the same kind of perforated plate.
